# Ineffective anti PD-1 therapy after BRAF inhibitor failure in advanced melanoma

**DOI:** 10.1186/s12885-018-4618-9

**Published:** 2018-07-03

**Authors:** M. Amini-Adle, N. Khanafer, M. Le-Bouar, G. Duru, S. Dalle, L. Thomas

**Affiliations:** 1 Department of Dermatology, Centre Hospitalier Lyon Sud, Hospices Civils de Lyon, Lyon 1 University, 165 Chemin du Grand Revoyet, 69495 Pierre Bénite Cedex, France; 2 Unit of Epidemiology and Infection Control Unit, Hôpital Edouard Herriot, Hospices Civils de Lyon, Laboratory of Emergent Pathogens, CIRI, Claude Bernard Lyon 1 University, Lyon, France; 3 Department of Biostatistics, Claude Bernard Lyon 1 University, 43 Bd 11 novembre 1918 BP 761, 69622 Villeurbanne Cedex, France

**Keywords:** Anti PD-1, BRAF inhibitor, Immunotherapy, Melanoma, Treatment sequence

## Abstract

**Background:**

Anti-PD-1 and BRAF-inhibitors (BRAFi) have been approved as first-line treatments in advanced melanoma. To date, no prospective data are available to give the best sequence of treatment. The objective of this study was to evaluate in real-life the efficacy of anti-PD-1 after BRAFi, ipilimumab, or chemotherapy failure.

**Methods:**

This was a single institution cohort analysis in patients treated with anti-PD-1 right after BRAFi, ipilimumab, or chemotherapy failure. Melanoma evolution after anti-PD-1 initiation was analyzed in BRAF-mutated and BRAF wild-type patients. The efficacy of treatment was evaluated by Objective Response Rate (ORR), Disease Control Rate (DCR), Progression-Free Survival (PFS), and Overall Survival (OS).

**Results:**

Seventy-four patients were included: 33 wild-type and 41 BRAF-mutated melanoma. ORR to anti-PD-1 was significantly lower in BRAF-mutated patients (12.2% vs. 45.5%, *p* = 0.002). After anti-PD-1 initiation, the median PFS and OS was significantly shorter in the BRAF mutated group (2 vs. 5 months and 7 vs. 20 months, *p* = 0.001). The hazard ratio for disease progression was of 2.3 (95%CI:1.3–3.9; *p* = 0.003) and 2.5 (95%CI:1.3–4.5; *p* = 0.005) for death. Thirty-nine percent of BRAF-mutated-patients died within 3 months after anti-PD-1 initiation. Rapid death (≤3 months) was significantly higher in BRAF-mutated patients (55.2% vs. 20.0%, *p* = 0.014).

**Discussion:**

This is the largest series of unselected patients treated in real-life with anti-PD-1 as second-or-higher line of treatment. Anti-PD-1 was less effective in BRAF-mutated cases as a majority of patients presented aggressive tumor evolution after BRAFi discontinuation. These data are consistent with previous studies suggesting a negative impact of BRAFi prior to immunotherapy.

## Background

Tyrosine kinase inhibitors and checkpoint inhibitor immunotherapies including anti CTLA-4 and anti PD-1 monoclonal antibodies have markedly improved prognosis of advanced melanoma [1]. In 2013, the society for immunotherapy published consensus recommendations for the use of immunotherapy in the management of advanced melanoma in the USA [2]. For BRAF-mutated patients, the authors suggest the use of immunotherapy first in patients with good performance status (PS). For patients with poor PS and brain metastasis the use of a BRAFi as first-line therapy was recommended. Recent European guidelines give less clear-cut recommendations for the management of BRAF-mutated metastatic melanoma patients, mentioning the possibility to use BRAFi in first or second-line therapy after anti-PD-1 failure [3]. To date, no randomized trial is available to provide evidence as to whether immunotherapy or BRAFi should be used first. Different studies suggest that the efficacy of BRAFi is not influenced by prior immunotherapy [4–7]. The efficacy of immunotherapy seems to be influenced by the aggressiveness and poor PS of patients who are not responding to BRAFi anymore [4–6]. Historically, anti-PD-1 therapy was approved after ipilimumab failure in BRAF wild-type metastatic melanoma and BRAFi failure in BRAF-mutated melanoma. Thereafter, anti-PD-1 obtained approval as first-line therapy in all stage IV melanoma patients according to phase-3 trials demonstrating the efficacy of anti-PD-1 regardless of BRAF status [8, 9]. To date, no prospective data is available regarding the efficacy of anti-PD-1 after BRAFi failure in real-life conditions of use. The objective of this study was to evaluate and compare the efficacy of anti-PD-1 in real-life use after progression during BRAFi or anti-CTLA-4 or chemotherapy in metastatic melanoma patients.

## Methods

An observational cohort study was conducted in a French university referral hospital (Lyon-Sud, Hospices Civils de Lyon). In accordance with the study objective, all unselected advanced melanoma patients treated between February 2013 and January 2017 with anti-PD-1 as second-or-higher line, regardless of the tumor burden, the PS status, or systemic steroid therapy for symptomatic brain metastasis, were included and followed until July 2017 (end of data collection). Patients initiating treatment after January 2017 were not included to allow at least 6 months follow-up. Mucosal melanoma and patients who were treated with anti-PD-1 due to toxicity of a previous line were excluded. Eligible patients were selected by searching in two prospective databases: Melbase and PAIR. Melbase is a French multicenter prospective database following patients starting first-line treatment for stage IV melanoma (NCT 02828202). PAIR (Programme d’Actions Intégrées de Recherche) is a single center cohort of patients treated with nivolumab as second line of therapy (NCT 02626065). All patients gave their consent to participate in these databases. These studies were approved by the ethical review board. The anti-PD-1 was administered immediately after previous line failure. Tumor assessments were made every 12 weeks from the time of administration of the first anti-PD-1 dose.

To measure efficacy, different parameters were evaluated, including Objective Response Rate (ORR), Disease-Control-Rate (DCR), Overall-Survival (OS), and Progression-Free-Survival (PFS). BRAF-mutated and wild-type melanoma patients were compared using these parameters.

To compare BRAF-V600 mutated and BRAF-wild-type patients, clinical, histopathological, and treatment characteristics were collected. The cohort was divided into two subgroups: BRAFV600 mutated and wild-type melanoma. Clinical and histopathological characteristics included age at diagnosis, gender, PS before first anti-PD-1 administration, presence of brain metastasis at initial stage IV disease and at first anti-PD-1 administration, number of metastatic sites (≤ or > 3 sites), and LDH level before anti-PD-1 treatment. Tumor characteristics included location, melanoma histopathological type, Breslow index, ulceration, and sentinel lymph node (SLN) status.

Treatment characteristics included number of lines and period of treatment before first anti-PD-1 administration and the number of patients treated with ipilimumab. Response to anti-PD-1 was evaluated according to the RECISTv1.1 criteria: ORR was calculated by the addition of complete (CR) and partial (PR) response. DCR was calculated by the addition of CR, PR, and Stable Disease (SD). PFS was the period of time from the first anti-PD-1 administration to first disease progression or death. OS was calculated from the first anti-PD-1 administration until death from any cause. Immune-mediated toxicities during anti-PD-1 therapy were collected.

### Statistical analysis

The distribution of continuous variables was verified. Chi-square or Fisher’s exact test (for qualitative variables) and the Mann-Whitney *U* test (for quantitative variables) were used to evaluate differences between patient subgroups. Kaplan-Meier analysis with the Log-rank test was used to estimate OS and PFS. Survival curves were used to estimate median of OS and PFS and Cox regression analysis was performed to estimate Hazard Ratios (HR) for disease progression or death. For all tests performed, 2-tailed *p* values < 0.05 were regarded as denoting statistical significance. Statistical data were analyzed Statistical Package for the Social Sciences (v17.0 for Windows, SECOG-PSS, Inc., Chicago, IL).

## Results

Seventy-four patients were included: 33 (44.6%) with wild-type-BRAF melanoma and 41 (55.4%) with BRAF-V600-mutated melanoma. The subgroups were well balanced in terms of age, sex, Breslow index, sentinel lymph node status and LDH level. The presence of brain metastasis at initial stage IV disease was significantly more frequent in the BRAF-mutated subgroup (*p* = 0.01). Before anti-PD-1 first administration, poor PS (≥1: 80.5% vs. 39.4%, *p* < 0.001), brain metastasis (61.0% vs. 18.2%, *p* < 0.001), and important tumor burden (> 3 metastatic sites: 48.8% vs. 24.2%; *p* = 0.04) were significantly higher in BRAF-mutated patients (Table 1).

**Table 1:** Characteristics of patients according to BRAF status

	Wild-type BRAF	Mutated BRAF	*p*
	*n* = 33	*n* = 41	
Demographic characteristics			
Median age at diagnosis, years [IQR]	59.4 [46.6–72.4]	51.3 [38.9–65.3]	0.10
Gender, *n* (%)			0.32
Male	23 (69.7)	24 (58.5)	
Female	10 (30.3)	17 (41.5)	
Clinical characteristics			
ECOG-PS ≥1, *n* (%)	13 (39.4)	33 (80.5)	< 0.001
Brain metastasis at initial stage IV disease, *n* (%)	3 (9.1)	14 (34.1)	0.01
Brain metastasis at first anti PD-1 administration, *n* (%)	6 (18.2)	25 (61.0)	< 0.001
Number of metastatic sites at anti PD-1 initiation			0.04
≤ 3, *n* (%)	16 (48.5)	17 (41.5)	
> 3, *n* (%)	8 (24.2)	20 (48.8)	
Elevated LDH before PD-1, *n* (%)	18 (54.5)	19 (46.3)	0.78
Tumor characteristics			
Known primary, *n* (%)	28 (84.8)	39 (95.1)	0.13
Location of primary			
Head and neck, *n* (%)	3 (10.7)	8 (20.5)	
Palms and soles, *n* (%)	5 (17.9)	0 (0)	
Upper and lower extremities, *n* (%)	11 (39.3)	16 (41.0)	
Trunk, n (%)	9 (32.1)	15 (38.5)	
Histopathological features			
Acrolentiginous melanoma, n (%)	7 (25.0)	0 (0)	
Desmoplastic, n (%)	2 (7.1)	0 (0)	
Nodular melanoma, n (%)	4 (14.3)	10 (25.6)	
Superficial Spreading Melanoma, n (%)	13 (46.4)	23 (59.0)	
Non classable, *n* (%)	2 (7.1)	4 (10.3)	
Breslow index, mm [IQR]	3.6 [1.8–4.8]	3.0 [1.8–4.2]	0.52
Ulceration, *n* (%)	16 (48.5)	17 (41.5)	0.04
Sentinel lymph node status, *n* (%)	15 (45.5)	17 (41.5)	0.26
Treatment characteristics			
Number of lines of therapy before anti PD-1			0.12
1, *n* (%)	21 (63.6)	36 (87.8)	
≥ 2, *n* (%)	12 (36.4)	5 (12.2)	
Period of systemic therapy before anti PD-1, months [IQR]	3 [3–11.5]	9 [6–20.5]	0.007
BRAF Inhibitor, *n* (%)		15 (36.6)	
BRAF + MEK inhibitors, *n* (%)		26 (63.4)	
Ipilimumab before anti PD-1, *n* (%)	31 (93.9)	5 (12.2)	
Anti PD-1 therapy			
Nivolumab, *n* (%)	22 (66.7)	36 (87.8)	
Pembrolizumab, *n* (%)	11 (33.3)	5 (12.2)	
Immune mediated toxicities, *n* (%)	15 (35.5)	4 (9.8)	0.003
Cutaneous rash, *n* (%)	5 (38.5)	0 (0)	
Diarrhea/enterocolitis, *n* (%)	1 (7.7)	2 (50)	
Hepatitis, *n* (%)	2 (15.4)	0 (0)	
Hypophysitis, *n* (%)	0 (0)	1 (25)	
Lung toxicity, *n* (%)	3 (23.1)	1 (25)	
Thyroiditis, *n* (%)	2 (15.4)	0 (0)	
Vitiligo, *n* (%)	7 (46.7)	2 (50)	
Other, *n* (%)	8 (61.5)	1 (25)	

Continuous variables are described by median and interquartile range and categorical variables by number and proportion

*Abbreviations*: *ECOG-PS* Eastern Cooperative Oncology Group Performance tatus, *LDH* lactate dehydrogenase, *PD-1* programmed cell Death-1s

Twelve of the 33 wild-type BRAF melanoma patients (36.4%) had at least 2 lines of treatment, and all except two received ipilimumab before anti-PD-1 administration (*n* = 31, 93.9%). Five BRAF-mutated patients received ipilimumab as first-line therapy followed by BRAFi. Before the first administration of anti-PD-1, BRAF-mutated patients had longer period of previous systemic therapy (median [interquartile range] 9 months [6–20.5] vs. 3 months [3–11.5]; *p* = 0.007). BRAFi alone was used in 15 patients (36.6%), and BRAFi combined with MEKi in and 26 patients (63.4%; Table 1). After BRAFi discontinuation, 39% (16/41) of patients died within 3 months. Rapid death (within 3 months) occurred more frequently in BRAF mutated patients after anti PD-1 initiation (55.2% (16/29) vs. 20.0% (4/20), *p* = 0.014). All patients died from disease progression.

### Response to anti-PD-1 according to BRAF status

**Fig. 1:**
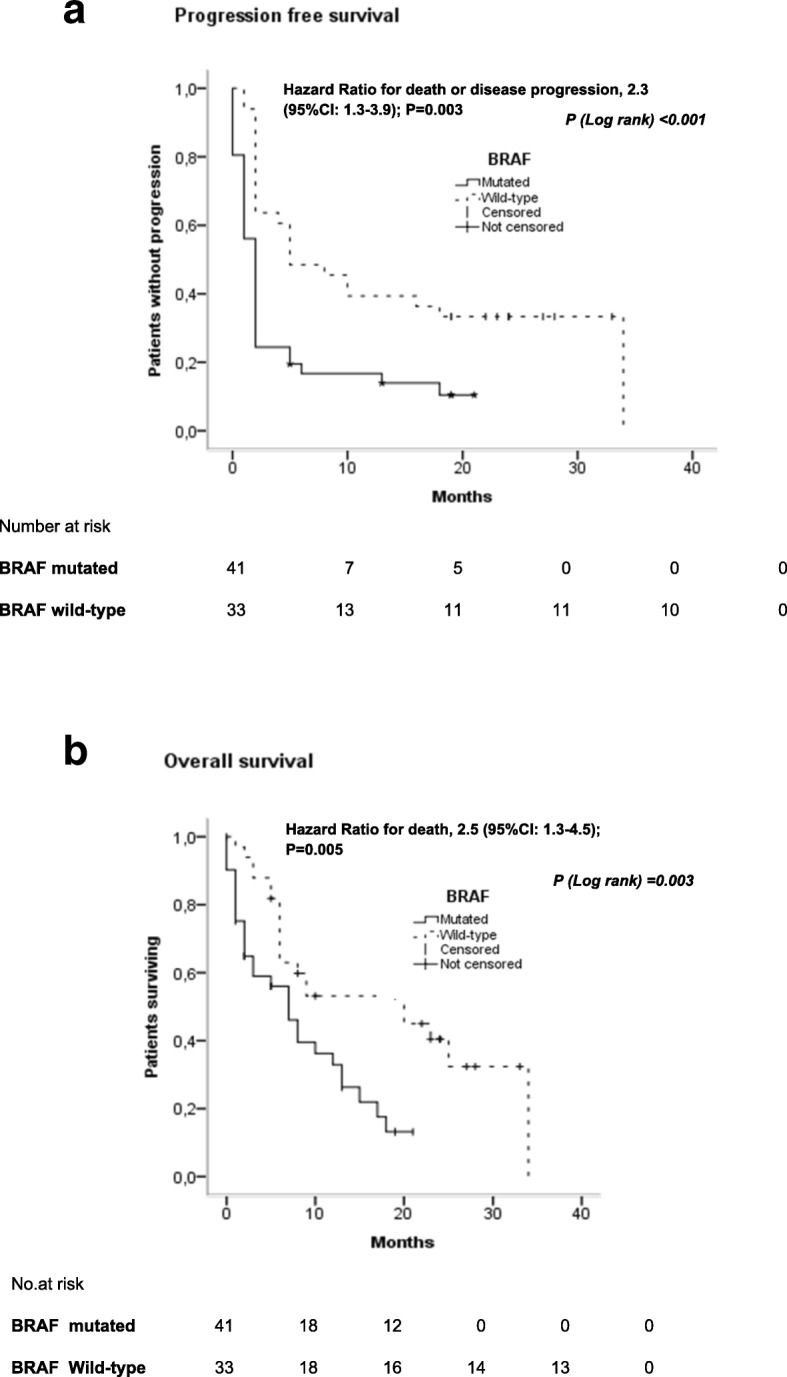
Survival analysis according to BRAF mutational status**. a** Kaplan Meier curve of Progression free survival (PFS) according to BRAF status. **b** Kaplan Meier curve of overall survival (OS) according to BRAF status

In the population as a whole, ORR to anti-PD-1 was 27.0%; DCR was 39.2% (11 CR, 9 PR and 9 SD). ORR (12.2% vs. 45.5%; *p* = 0.003) and DCR to anti-PD-1 (24.4% vs. 57.6%; *p* = 0.008) was significantly lower in BRAF-mutated patients compared to wild-type patients (Table 2). All BRAF-mutated responders (3 CR and 2 PR) were free of brain metastasis at initial stage IV diagnosis; 4 had ≤3 metastatic sites and PS = 0. From initiation of anti PD-1, the median PFS was 2 months (95% CI, 1.6–2.4) in the BRAF mutated group and 5 months (95% CI, 0.2–9.8) in the wild BRAF group (Table 2). A significant benefit with respect to PFS **and OS** was observed for wild-type BRAF patients as compared to those with mutated BRAF (HR for disease progression or death was 2.3; 95%CI, 1.3–3.9; *p* = 0.003, Fig. 1a; HR for overall survival was 2.5; 95%CI, 1.3–4.5; *p* = 0.005, Fig. 1b). OS was 20 months (95%CI, 5.4–34.6) in patients with wild-type BRAF and 7 months (95%CI, 4.0–10.0) in those with mutated BRAF (Table 2). A significant benefit with respect to OS was observed in patients with wild-type BRAF as compared to those with mutated BRAF (HR for death, 2.5; 95%CI, 1.3–4.5; *p* = 0.005).

**Table 2:** Response to anti PD-1 according to BRAF status

	Wild-type BRAF	Mutated BRAF	*p*
	*n* = 33	*n* = 41	
Objective Response Rate, *n* (%)	15 (45.5)	5 (12.2)	0.003
Disease Control Rate, *n* (%)	19 (57.6)	10 (24.4)	0.008
Progressive Disease, *n* (%)	14 (42.4)	31 (42.4)	
Median Progression-Free Survival, months [95%CI]	5 [0.2–9.8]	2 [1.6–2.4]	< 0.001
Median Overall Survival, months [95%CI]	20 [5.4–34.6]	7 [4.0–10.0]	< 0.001

*CI* confidence interval

### Toxicities to anti-PD-1

There were 7 cases (11.7%) of grade 3–4 toxicity, including two cases of interstitial pneumonia, two cases of uveitis, one case of colitis, one case of hepatitis; and one case of hypophysitis. Immune-mediated toxicities occurred in 19 patients (25.7%), the most frequent was vitiligo (*n* = 9, 12.2%), followed by skin rash (*n* = 5, 6.8%) lung toxicity (*n* = 4, 5.4%), thyroiditis (*n* = 2, 2.7%), colitis (*n* = 2, 2.7%) and hepatitis (*n* = 2, 2.7%). Immune mediated toxicities were more frequent among wild-type BRAF patients than those with mutated BRAF (35.5% vs. 9.8%, *p* = 0.003).

## Discussion

We report here the largest retrospective series of unselected patients treated in real-life during the period when anti-PD-1 was only allowed in patients previously treated by anti CTLA-4 or BRAFi according to BRAF status. While the standard of care has changed since this period, with the approval of anti-PD-1 as first line of treatment regardless the BRAF status, the data of the present study confirm previous recent retrospective studies demonstrating the lack of efficiency of anti-PD-1 after BRAFi failure [10, 11]. For instance, in a study reported by Simeone et al. patients with BRAF-mutated melanoma previously treated with BRAFi had a significantly lower median PFS (3 months vs. not reached) and DCR (18.6% vs. 65.4%) to pembrolizumab compared to wild-type melanoma treated with anti-PD-1 after ipilimumab escape [10]. These studies reflect the real-life treatment of the target population, and the results herein also suggest aggressive melanoma progression after BRAFi discontinuation. Poorer PS, more frequent brain metastasis, and higher tumor burden are the factors that preclude any benefit from subsequent immunotherapy and explain the high death rate (almost 40%) within 3 months following BRAFi discontinuation.

The aggressive evolution found in the present study is concordant with both phase 2 (BRIM-2) [12] and phase 3 (BRIM-3) [13] trials of vemurafenib in which 41 and 52% patients, respectively, died in the month following vemurafenib discontinuation. Other retrospective studies have also confirmed poor OS or rapid progression after BRAFi interruption [4, 6, 7]. In these studies, rapid progressive disease after BRAFi failure was found to be associated with high LDH [4, 6], brain metastasis [4, 6], poor PS [4, 6], and younger age [6]. Ascierto et al. studied a large Italian retrospective series of 93 BRAF-mutated patients treated either with BRAFi first (*n* = 45) or ipilimumab first (*n* = 48) [7]. The authors found a better OS in patients treated with immunotherapy first (14.5 vs. 9.9 months, *p* = 0.04). After BRAFi, 40% were rapid progressors and were unable to complete 4 courses of ipilimumab. However, caution should be taken when interpreting these results as patients without brain metastasis and normal LDH were selected to receive immunotherapy first.

Taken together, our results corroborate these retrospective data suggesting the lower efficacy of immunotherapy after BRAFi failure. These data are important to take into account, pending the results from an ongoing large phase-3 clinical trial (NCT02224781) testing two arms of BRAF-mutated patients first treated with BRAFi and MEKi followed by combination immunotherapies (ipilimumab + nivolumab) or the reverse sequence.

Currently immuno-oncology recommendations consider treating patients with high tumor burden, brain metastasis, and clinically symptomatic disease with BRAFi first [2]. For other patients, the present study and the aforementioned retrospective studies suggest that the use of immunotherapy first is associated with improved clinical benefit.

However, other studies did not demonstrate a negative impact of previous treatment with BRAFi to subsequent immunotherapy. For instance, a recent small retrospective series of BRAF-mutated patients compared two groups of populations receiving a sequence of BRAFi followed by immunotherapy (*n* = 16) or the reverse sequence (*n* = 9) [14]. ORR achieved by BRAFi was not different between groups. No difference was found in terms of OS between study groups, and a higher response rate to immunotherapy was observed in patients treated with immunotherapy after BRAFi (43.8% vs. 0%). Furthermore, in a retrospective analysis of data pooled from 4 clinical trials analyzing 440 patients, 334 wild-type and 106 BRAF-mutated patients, the ORR did not seem to be affected by prior BRAFi or ipilimumab therapy. The ORR was 33.1% in BRAF-mutated patients naïve to BRAFi, and 24.5% in patients who had received prior BRAFi [15]. However, these results cannot be extrapolated to a real-life situation as they are pooled from clinical trials and overestimate nivolumab activity in BRAF patients who were selected for the purposes of the trial without brain metastasis or poor PS.

The present study is limited due to its retrospective non-randomized single institution nature. The small sample size of this cohort did not allow multivariate analysis giving less powerful results. The analysis is further limited by the lack of an analysis of mutational burden, which is known to correlate with response to checkpoint inhibitors.

Taken together, data currently available in the literature are contradictory as to which sequence of treatment is best for BRAF-mutated patients. However, in real life use, we found that the aggressiveness of melanoma progression after BRAFi interruption represents an important factor which could precipitate failure of subsequent immunotherapy. These results deserve to be confirmed by larger prospective cohorts of patients receiving anti-PD-1 in real-life use. Such studies would also allow the determination of factors associated with immunotherapy failure.

## Conclusions

In summary, we report here the results from a large retrospective of unselected patients treated in real-life-use with anti PD-1 after chemotherapy-ipilimumab or BRAFi failure. We found significant difference in term of ORR, PFS, and OS between BRAF mutated and BRAF wild type patients. Patients previously treated with BRAFi had more aggressive tumor evolution precipitating failure of subsequent anti PD-1.

## Abbreviations

BRAFi: BRAF inhibitor
CR: Complete Response
CTLA-4: Cytotoxic T-lymphocyte–associated antigen-4
DCR: Disease Control Rate
HR: Hazard Ratio
MEKi: MEK inhibitor
ORR: Objective Response Rate
OS: Overall Survival
PD-1: Programmed Death-1
PFS: Progression Free Survival
PR: Partial Response
PS: Performance Status
SD: Stable Disease
